# An Integrated Analysis of Intracellular Metabolites and Virulence Gene Expression during Biofilm Development of a Clinical Isolate of *Candida tropicalis* on Distinct Surfaces

**DOI:** 10.3390/ijms22169038

**Published:** 2021-08-21

**Authors:** Maria Michela Salvatore, Angela Maione, Luisa Albarano, Elisabetta de Alteriis, Federica Carraturo, Anna Andolfi, Francesco Salvatore, Emilia Galdiero, Marco Guida

**Affiliations:** 1 Department of Chemical Sciences, University of Naples ‘Federico II’, Via Cinthia, 80126 Naples, Italy; mariamichela.salvatore@unina.it (M.M.S.); andolfi@unina.it (A.A.); frsalvat@unina.it (F.S.); 2 Department of Biology, University of Naples ‘Federico II’, Via Cinthia, 80126 Naples, Italy; angela.maione@unina.it (A.M.); luisa.albarano@unina.it (L.A.); dealteri@unina.it (E.d.A.); federica.carraturo@unina.it (F.C.); marco.guida@unina.it (M.G.); 3 BAT Center—Interuniversity Center for Studies on Bioinspired Agro-Environmental Technology, University of Naples ‘Federico II’, 80055 Naples, Italy

**Keywords:** biofilm, *Candida tropicalis*, medical devices, metabolomics, gene expression

## Abstract

Emergence of *Candida tropicalis*, which causes potential life-threatening invasive candidiasis, is often associated with colonization of medical devices as biofilm. Biofilm plays an important role in the virulence of the pathogen because of its complex structure, which provides resistance to conventional antimicrobials. In this study, the metabolic response of a clinical strain of *C. tropicalis* colonizing three distinct surfaces (polytetrafluoroethylene (PTFE), polystyrene, and polycarbonate) as well as the expression of virulence and stress related genes (*ALS3, Hsp21*, *SAP1*, *SAP2*, *SAP3*, and *CYR1*), were explored. Our results showed that lesser biofilm was developed on PTFE compared to polystyrene and polycarbonate. GS-MS metabolic analysis identified a total of 36 metabolites in the intracellular extract of cells grown on polystyrene, polycarbonate, and PTFE, essentially belonging to central carbon metabolism, amino acids, and lipids metabolism. The metabolic analysis showed that saturated and unsaturated fatty acids are preferentially produced during biofilm development on polycarbonate, whereas trehalose and vitamin B6, known as cellular protectors against a variety of stressors, were characteristic of biofilm on PTFE. The results of the transcriptomic analysis consider the different degrees of colonization of the three substrates, being *CYR1*, which encodes the component of signaling pathway of hyphal formation-cAMP-PKA, downregulated in PTFE biofilm compared to polycarbonate or polystyrene biofilms, while *Hsp21* was upregulated in concomitance with the potential unfavorable conditions for biofilm formation on PTFE. Overall, this work provides new insights into the knowledge of *C. tropicalis* biofilm development on surfaces of medical relevance in the perspective of improving the management of *Candida* infections.

## 1. Introduction

*Candida* species are the most common cause of opportunistic fungal infections, mainly in immunocompromised individuals. If *Candida albicans* is still the most commonly isolated species, other non-albicans *Candida* species such as *Candida tropicalis* have been isolated from patients and have attracted attention because of their resistance to antifungals. A major characteristic of *C. tropicalis* infections is the formation of biofilms on different surfaces of medical devices and implants such as catheters, artificial heart valves, maxillofacial prostheses, pacemakers, prosthetic joints, and contact lenses that pose serious risks for functional, medical, and esthetic problems [1].

Biofilm mainly consists of micro-colonies of yeast, hyphae, pseudo hyphae, and extra-cellular matrix arranged in a complex structure, conferring significant tolerance to antifungal therapy by limiting the penetration of substances through the biofilm matrix and protecting the embedded cells from host immune responses and enhancing pathogenicity.

The frequency of *C. tropicalis* has increased from 40% to 70% of mortality caused by blood infections, found in neutropenic patients and individuals with hematological malignancies and extended time in intensive care units [2].

The pathogenicity of *C. tropicalis* is therefore linked to its great ability to adhere to different materials, forming biofilm with a different degree of virulence and the capacity to secrete hydrolytic enzymes containing aspartyl proteases, phospholipases, and hemolysins. A large number of genes are affected in their expression when an organism responds to an environmental stress such as the switching to a hyphal form.

Adhesion, biofilm formation, and hydrolytic enzyme activity were recognized as the key pathogenic elements of *Candida* species. Previous studies on *C. tropicalis* reported that *SAP* genes, which encode for secreted aspartic proteases (Saps), are a crucial virulence attribute, as well as *ALS3* (which is one of the 16 members of the *ALS* family) and *CYR1*. In fact, *ALSs* are hyphae-specific genes that encode cell wall proteins that are important in the adhesion to host cells and *CYR1* promotes the activation of cAMP-responsive protein kinase A subunits and increases transcription of genes involved in or associated with hyphal growth. The *Hsp21* family is crucial for *Candida* cells during exposure to stressors encountered during pathogenesis [3,4,5].

Previous studies have demonstrated that *Candida* can readily attach and form biofilms in vivo and in vitro on medically relevant surfaces and that hydrophobic materials were those with the highest biofilm formation [6].

In this work, we analyze biofilm formation of *C. tropicalis* on three different materials: polystyrene, polycarbonate, and polytetrafluoroethylene (PTFE). Polystyrene is an important surface in the medical field. Due to its inertness and biocompatibility, it is widely used for the production of biomedical devices and laboratory equipment [7]. PTFE, popularly known as the commercial name Teflon^®^, together with polycarbonate, are hydrophobic biocompatible materials which are current components of medical and surgical devices widely used in cardiovascular, neuro-, and vitreoretinal surgery [8].

All three materials can impact fungal adhesion and biofilm growth during healthcare-associated infections.

Microbial metabolomics is a comprehensive analysis of metabolites produced by microorganisms as final products of biochemical processes and results of environmental and genetic interactions. Metabolomics provide a comprehensive representation of metabolites and shows further insights into the mechanisms involved in stress responses during infectious diseases.

Exploring the relationship between the genotypes and metabolomics results can be a new approach for *C. tropicalis* pathogenicity studies.

Thus, for a better knowledge of *C. tropicalis* biofilms in the perspective to improve the treatment for nosocomial diseases, it may be of significant necessity to characterize the dynamic metabolic changes associated with biofilm formation on different biomaterials and to find out their molecular differences.

Therefore, the purpose of this study is to investigate metabolomics profiling during building biofilms and to correlate specific metabolites concerned in the complex development process of biofilm formation with certain virulence-related genes. The results of this integrated analysis may be useful for exploring the relationship between metabolomics and gene expression in *C. tropicalis* during biofilm formation on the different materials tested.

In this study, we focus on results obtained using a *C. tropicalis* strain isolated from systemic infection, which is briefly denoted as *C. tropicalis* clinical strain. However, most experiments were extended to *C. tropicalis* DSM 11951 strain, which acts as a control to support or verify the experimental and data analysis processes.

## 2. Results

### 2.1. Biofilm Formation

**Figure 1:**
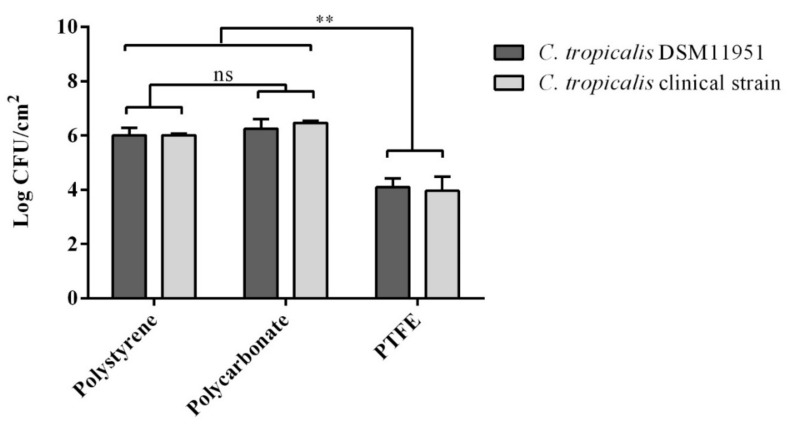
Log fungal density (means and standard deviations) of *C. tropicalis* DSM 11951 and *C. tropicalis* clinical isolate for each material evaluated. ns: not significant, ** *p* value < 0.01 (Tukey’s).

First, we evaluated the capability of the two *C. tropicalis* strains to form biofilms on abiotic surfaces enumerating colony forming units (CFUs). As shown in Figure 1, both strains were equally able to form biofilms on the three materials tested. However, compared to polystyrene and polycarbonate, significantly less biofilm was developed on PTFE on which fungus cell density decreased about one hundred times. The different composition, porosity, hydrophobicity, and irregularities of the three surfaces can be invoked to rationalize the results obtained, but phenomenologically, it is evident that PTFE surface is less suitable for biofilm formation and growth or, vice versa, that *C. tropicalis* biofilms are most efficiently established on polycarbonate or polystyrene surfaces.

### 2.2. GC-MS Analysis of Intracellular Metabolites

Biofilms were grown for 72 h on three different substrates (i.e., polystyrene, polycarbonate, and PTFE) in order to compare their metabolic profiles. To reach this goal, intracellular metabolites were extracted from cells and, after derivatization with *N*,*O*-Bis(trimethylsilyl)trifluoroacetamide (BSTFA), were submitted to GC-MS analysis. Overall, two datasets (one dataset for each strain) were collected.

A dataset incorporates three classes or conditions corresponding to the three adhesion materials investigated and identified with labels PolyStyr, PolyCarb, and PTFE. Each class comprises six GC-MS data files (GC-MS chromatograms), representing three technical replicates for each of two biological replicates under identical acquisition and cultural conditions.

The GC-MS datafiles were processed with the NIST program AMDIS for deconvolution, component detection, and area peak determination. AMDIS results files, segmented according to the three classes, were presented to SpectConnect program, which performs correspondence analysis and matches components within and between classes. Furthermore, SpectConnect creates a relative abundance matrix (RA matrix) with rows corresponding to observations and columns to “conserved metabolites”, which is the basis of the following evaluations [9]. In the context of SpectConnect software, a conserved metabolite is one whose signal consistently persists in technical and biological replicates under identical conditions. The concept of conserved metabolite, which will be repeatedly used in the following, is further expanded in Materials and Methods.

#### 2.2.1. Multivariate Data Analysis

The two RA matrices (one for each strain) provided by the pre-processing steps were submitted to multivariate analysis to find if significant differences exist between the two datasets. To this end, the RA matrix was imported in our in-house MATLAB .m script [10], which has the logic for performing principal component analysis (PCA) and partial least square discriminant analysis (PLS-DA) multiclass comparisons. To make metabolites equally important, before multivariate analysis, the RA matrix is mean centered and variance scaled.

**Figure 2:**
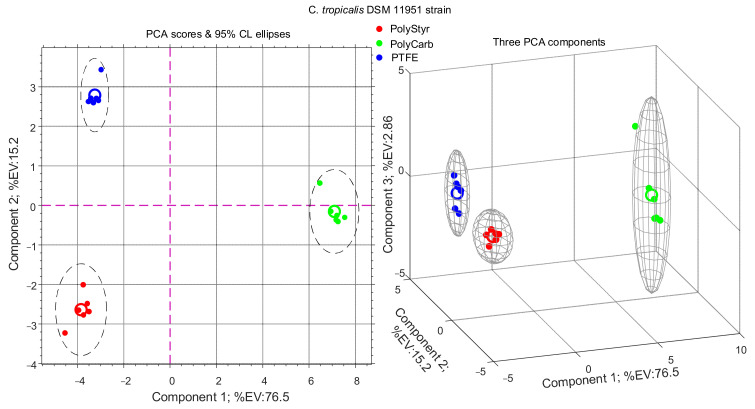
PCA analysis of intracellular metabolites extracted from cells of *Candida tropicalis* DSM 11951 strain harvested from biofilms grown (72 h) on polystyrene (PolyStyr class), polycarbonate (PolyCarb class), and Teflon (PTFE class).

**Figure 3:**
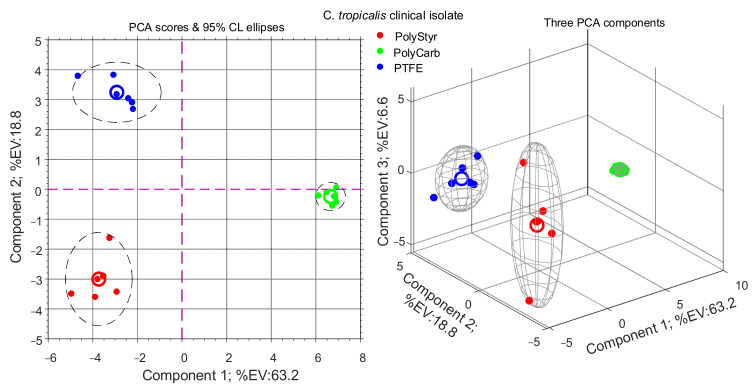
PCA analysis of intracellular metabolites extracted from cells of the clinical isolate of *Candida tropicalis* harvested from biofilms grown (72 h) on polystyrene (PolyStyr class), polycarbonate (PolyCarb class), and Teflon (PTFE class).

Results of PCA analysis for the two datasets considered in this study are presented in Figure 2 and Figure 3 in the form of PCA scores scatter plots.

From Figure 2 and Figure 3, it appears that PolyCarb class is separated along the principal PCA axis from PolyStyr and PTFE classes. On the contrary, PolyStyr and PTFE classes share the same principal component coordinate, although they are separated along the second component axis, which however accounts for only about 15–18% of the total variance. This may be interpreted to signify that metabolites levels in cells from biofilms grown on polystyrene and PTFE are considerably similar to each other and differ substantially from metabolite levels in cells from biofilms grown on polycarbonate.

Because both strains of *C. tropicalis* investigated in this study display the same class pattern, in the following we will focus on the clinical isolate dataset.

**Figure 4:**
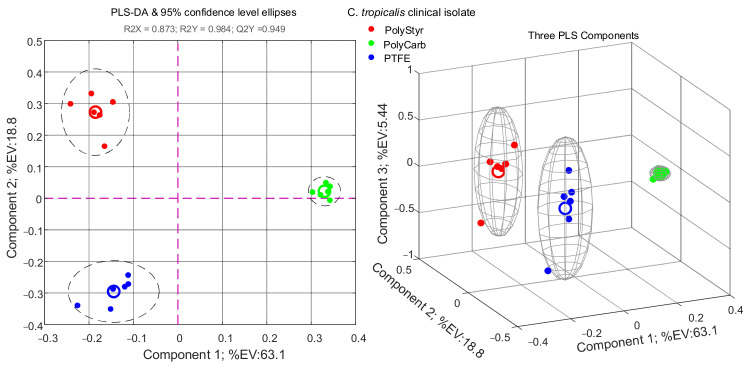
PLS-DA analysis of intracellular metabolites extracted from cells of the clinical isolate of *Candida tropicalis* harvested from biofilms grown (72 h) on polystyrene (PolyStyr class), polycarbonate (PolyCarb class), and Teflon (PTFE class).

As a complement to the PCA analysis in Figure 3, PLS-DA is a convenient supervised multivariate data analysis technique since it allows the ranking of variables (metabolites) on the basis of their variable importance for projection (VIP) score. Variables with VIP values greater than 1 are considered important for grouping and separation between classes in the PLS-DA model. The results of the PLS-DA analysis of the clinical isolate dataset are presented in Figure 4 and Table 1.

The PLS-DA model in Figure 4 was validated by the statistics: R2X = 0.873; R2Y = 0.984; and Q2Y = 0.949. The high value of Q2Y, calculated using mean squared errors from fivefold cross-validation, indicates that the PLS-DA model is a predictive one.

As can be seen from Figure 4, the PLS-DA model retains the essential traits of the PCA model in Figure 3. However, as expected, considering that PLS-DA technique is supervised and class information is known ex ante and used to develop the model, a better separation of the PolyStyr and PTFE classes along the PLS-DA component 2 is obtained.

In Table 1, besides the VIP score obtained from PLS-DA analysis, an ID_## string, which will serve, when appropriate, as a shortcut for the metabolite full name, is associated to each identified metabolite. Furthermore, for each identified metabolite, in the column labeled “In Class”, an array of digits that shows the classes in which the metabolite was found to be conserved, is included.

**Figure 5:**
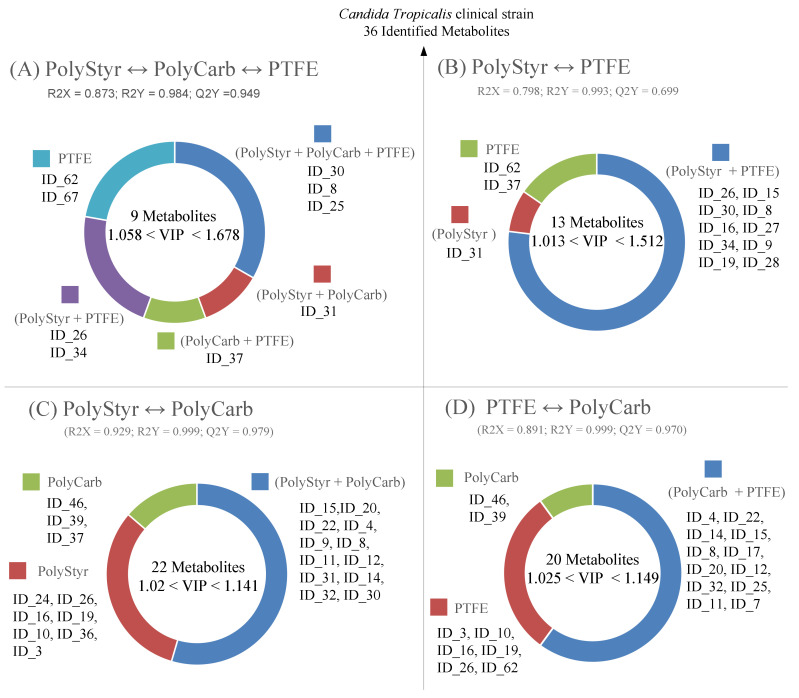
Visual presentation of results of interclasses PLS-DA analyses (**A**–**D**). Only the set of metabolites with VIP scores > 1, which significantly contributes to the PLS-DA models, is presented. PLS-DA models have been validated by the statistics R2X, R2Y, and Q2Y (from five-fold cross validation), whose values are reported for each model in the figure. Metabolites can be tracked back to identified metabolites in Table 1 through the ID_## string.

By relying on the concept of “conserved metabolite”, as used in the context of SpectConnect software, we can gain an understanding of the reasons why a VIP score of greater than one is assigned to a given metabolite within the PLS-DA model of Figure 4. As shown in Figure 5A, metabolites in Table 1 with VIP scores greater than 1 can be collected under one of five labels, each label defining the class or classes within which a given metabolite, identified by its ID_## string, is found to be conserved. For instance, under the label PolyStyr + PolyCarb + PTFE, there are reported metabolites that are found to be conserved in all three classes (conditions) and under the label PolyStyr + PTFE, there are collected metabolites that are found to be conserved only in the PolyStyr and PTFE classes and are not detected in the PolyCarb class. Figure 5A shows how the nine metabolites with VIP score > 1 in Table 1 are distributed under the five labels.

The fundamental idea is that when a metabolite is conserved in replicated samples of class 1, but it is not conserved or detected in samples of class 2, it means that this metabolite is upregulated in class 1 with respect to class 2 or, alternatively, that this metabolite is downregulated in class 2 with respect to class 1.

By consequence, from Figure 5A, we observe that the metabolite identified by ID_62 (corresponding to trehalose in Table 1) is upregulated in cells from biofilms grown on PTFE (PTFE class), both with respect to cells from biofilms grown on polycarbonate (PolyCarb class) and on polystyrene (PolyStyr class). Similarly, the metabolite with ID_37 (corresponding to succinic acid) is upregulated both in the PolyCarb and PTFE classes with respect to PolyStyr class. Conversely, if we consider the metabolite with ID_34 (corresponding to lysine), we can observe that lysine is downregulated in PolyCarb class both with respect to PolyStyr and PTFE classes.

Then, a wealth of information on the relative levels of the nine metabolites that significantly contribute to the PLS-DA model, as seen in Figure 4, in cells of the three different biofilms, is incorporated in Figure 5A from which one can obtain an understanding on why specified metabolites significantly contribute to the separation between classes.

Nevertheless, little can be deduced from Figure 5A concerning the distribution of the three metabolites with ID_8, ID_25, and ID_30 (corresponding to phosphoric acid, tyrosine, and 1-monopalmitine, respectively) because they are conserved in all three classes. Excepting that these metabolites are differently distributed in cells of the three biofilms because they have VIP scores > 1 and contribute significantly to the PLS-DA model.

Further differences and similarities between different conditions can be detected by PLS-DA comparisons between class pairs. The results of the three possible PLS-DA pairwise comparisons are presented in Figure 5B–D (by leveraging, as in Figure 5A, on the concept of conserved metabolite).

As noted above, the level of intracellular metabolites in cells from biofilms grown on polystyrene and on PTFE are similar because PolyStyr and PTFE classes are not separated on the principal component axis of the PCA scores plot of Figure 3. Figure 5B confirms the similarities between these two classes. This can be seen because of the 13 metabolites that significantly contribute to the PLS-DA model, 10 are conserved in both classes.

Analogously, Figure 5C,D provides understanding for the differences (which were already brought to light by the PCA analysis in Figure 3) between PolyCarb class on one side, and PolyStyr and PTFE classes on the other side. PLS-DA comparisons of PolyCarb class vs. PolyStyr (Figure 5C) or vs. PTFE (Figure 5D) classes produce models in which significant differences between pairs of classes are spread on a large number of metabolites with VIP scores moderately exceeding one, and it is noteworthy that the donut charts in Figure 5C,D are remarkably similar.

#### 2.2.2. Univariate Data Analysis

**Figure 6:**
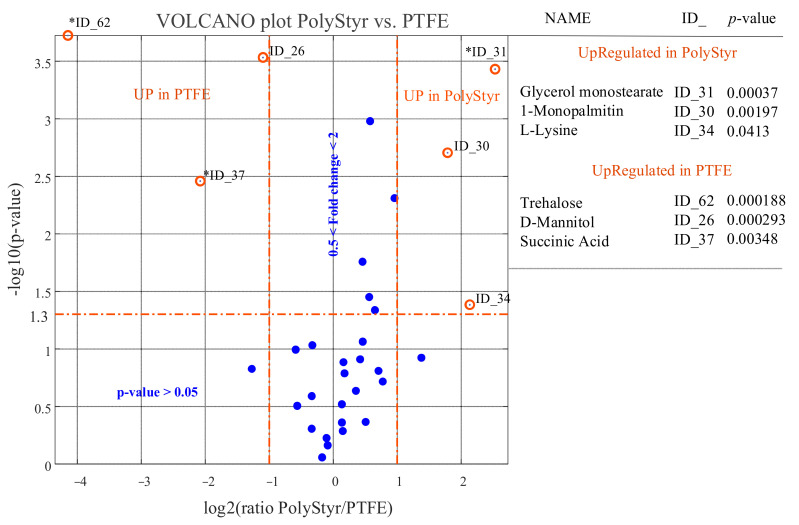
Results of univariate comparison of polystyrene (PolyStyr class) vs. Teflon (PTFE class). For each metabolite, fold change (FC) is defined as the ratio between mean relative abundances in PolyStyr and PTFE classes. Only metabolites with *p* value < 0.05 and fold changes greater than 2 are considered upregulated in the PolyStyr class, and only metabolites with *p* value < 0.05 and fold changes lower than 0.5 are considered upregulated in PTFE class (orange rings). Blue dots represent metabolites with no significantly different levels in the two compared classes. Fold change of metabolites marked with an asterisk (*) has been evaluated by the data imputation strategy described in the text. Metabolites can be tracked back to identified metabolites in Table 1 through the ID_## string.

Univariate analysis of data was performed in order to compare, one by one, metabolites levels in any pair of classes (conditions). Results of the univariate comparison of the PolyStyr and PTFE classes, for the 32 identified metabolites that are conserved in at least one of the two classes, are exposed in Figure 6 and in Table S1.

As can be seen from the volcano plot in Figure 6, univariate analysis associates to each metabolite a fold change (FC) that is defined as the ratio between the average of the 6 × 1 vector of relative abundances representing the metabolite in the PolyStyr class and the average of the 6 × 1 vector of relative abundances representing the metabolite in the PTFE class. In abstract, the fold change can be interpreted as expressing the ratio of the amount of a metabolite in single cells of *C. tropicalis* from biofilm grown on polystyrene to its amount in cells from biofilm grown on PTFE.

However, the calculated fold changes need to be validated for statistical significance. This is accomplished by associating, to each calculated fold change, a *p* value, which is calculated by submitting the two 6 × 1 vectors of relative abundances, from which the FC is calculated, to a Student’s *t*-test for equal means. Only metabolites with a *p* value < 0.05 and fold changes greater than 2 are considered to be upregulated in the PolyStyr class, and only metabolites with a *p* value < 0.05 and fold changes lower than 0.5 are considered to be upregulated in the PTFE class (or downregulated in the PolyStyr class).

While the fold change calculation is straightforward for metabolites that are conserved in both compared classes, problems are encountered for metabolites that are observed only in one of the two classes. This is because one of the two compared vectors is a vector of all zeros so that an infinite (Inf)- or zero-fold change, and a point with a ± Inf abscissa in the volcano plot, will be obtained. This difficulty can be prevented by a data imputation strategy implemented on the RA matrix. A reasonable data imputation strategy may consist in substituting all zeros in a row of the RA matrix with a fraction of the minimum relative abundance value in the row. This is equivalent to the attribute, to the undetected peak of an expected metabolite in a chromatogram, an area equal to a fraction of the area of the smallest peak detected.

In the volcano plot of Figure 6, the large prevalence of blue dots (representing metabolites that have no statistically significant different levels in the two compared conditions or for which 0.5 < FC < 2) is further evidence that few differences in intracellular metabolites levels exist between *C. tropicalis* biofilms grown on polystyrene or on PTFE surfaces. Only three metabolites are upregulated in the PolyStyr class and only three metabolites are upregulated in the PTFE class. This result is consistent with what can be deduced from Figure 5B.

**Figure 7:**
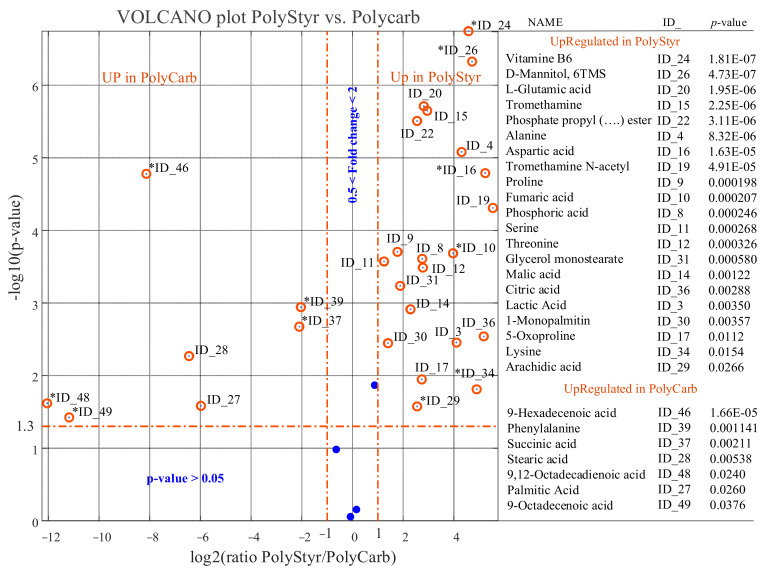
Results of univariate comparison of polystyrene (PolyStyr class) vs. polycarbonate (PolyCarb class). For each metabolite, fold change (FC) is defined as the ratio between mean relative abundances in PolyStyr and PolyCarb classes. Only metabolites with *p* value < 0.05 and fold changes greater than 2 are considered upregulated in the PolyStyr class, and only metabolites with *p* value < 0.05 and fold changes lower than 0.5 are considered upregulated in PolyCarb class (orange rings). Blue dots represent metabolites with no significantly different levels in the two compared classes. Fold change of metabolites marked with an asterisk (*) has been evaluated by the data imputation strategy described in the text. Metabolites can be tracked back to identified metabolites in Table 1 through the ID_## string.

**Figure 8:**
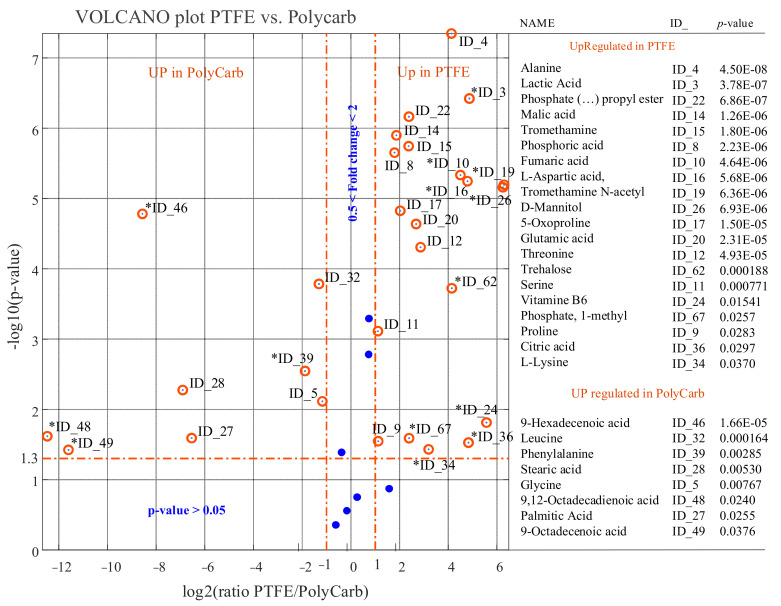
Results of univariate comparison of Teflon (PTFE class) vs. polycarbonate (PolyCarb class). For each metabolite, fold change (FC) is defined as the ratio between mean relative abundances in PTFE and PolyCarb classes. Only metabolites with *p* value < 0.05 and fold changes greater than 2 are considered upregulated in the PTFE class, and only metabolites with *p* value < 0.05 and fold changes lower than 0.5 are considered upregulated in PolyCarb class (orange rings). Blue dots represent metabolites with no significantly different levels in the two compared classes. Fold change of metabolites marked with an asterisk (*) has been evaluated by the data imputation strategy described in the text. Metabolites can be tracked back to identified metabolites in Table 1 through the ID_## string.

We see from Figure 7 and Figure 8 that the scenario changes dramatically both when the level of intracellular metabolites in cells from biofilms grown on polystyrene or on PTFE is compared with intracellular metabolite levels in cells from biofilms grown on polycarbonate.

A reduced number of identified metabolites, represented by blue dots, have comparable levels in PolyStyr or PTFE classes and the PolyCarb class.

### 2.3. Comparative Quantitation of Virulence and Stress Response Genes Expression

In order to study the impact of distinct materials on biofilm formation, the expression of six selected genes (i.e., *ALS3*, *Hsp21*, *SAP1*, *SAP2*, *SAP3*, and *CYR1*) involved in the virulence or stress response of *C. tropicalis* was investigated on biofilms developed for 72 h on the three different substrates (i.e., polystyrene, polycarbonate, and PTFE).

The expression of each gene was evaluated by real-time qRT-PCR. Comparative quantification of gene expression in cells from biofilms grown on two different surfaces was performed by the 2(-DELTA DELTA) Ct method (i.e., by evaluating the fold change from the formula: $\mathrm{fold}\;\mathrm{change}\;=2^{-\Delta \Delta \mathrm{Ct}}$) [11]. Ct represents the fractional cycle number at which the fluorescence passes the threshold and the housekeeping *ACT1* gene is used for normalization.

For each gene in each comparison, an average fold change was evaluated from three replicate experiments and submitted to a one-sample *t*-test to assess whether it was statistically different from 1.

**Figure 9:**
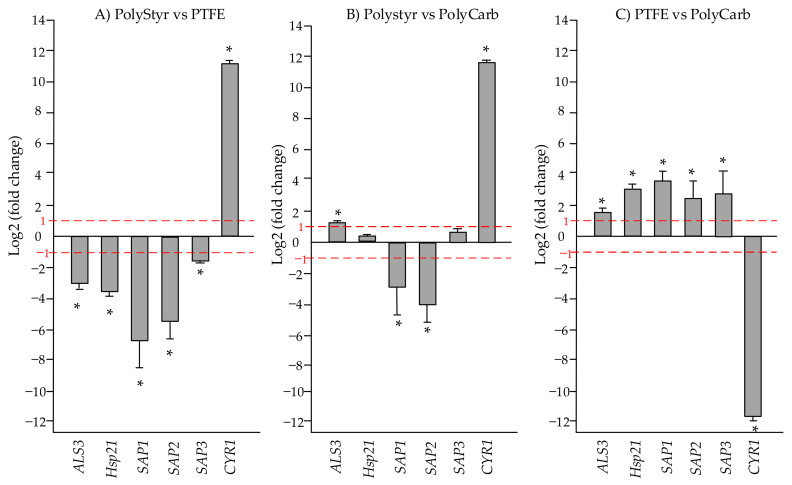
Mean relative mRNA expression levels of virulence and stress response genes of clinical isolate of *C. tropicalis*. (**A**) Polystyrene vs. polytetrafluoroethylene (PTFE); (**B**) polystyrene vs. polycarbonate; (**C**) PTFE vs. polycarbonate. Fold changes are calculated according to the formula $\mathrm{fold}\;\mathrm{change}\;=2^{-\Delta \Delta \mathrm{Ct}}$ by using Ct (threshold cycle number) generated by the qRT-PCR system. Reported fold changes are the means of three replicate experiments. Fold changes significantly different from 1 (*p* value < 0.05) are marked by an asterisk. Red broken lines indicate fold change thresholds of 2 and 0.5 respectively.

Results of the three possible pairwise comparisons of gene expression levels in cells from biofilms of the clinical strain of *C. tropicalis* grown on polystyrene, polycarbonate, and PTFE surfaces are exposed in Figure 9. Only fold changes larger than 2 (i.e., log2(fold change) > 1) or lower than 0.5 (i.e., log2(fold change) < −1) and for which the one-sample *t*-test restituted a *p* value lower than 0.05 are considered significantly different from 1.

From Figure 9A,C, in cells from biofilms on PTFE, *CYR1* gene is downregulated and all other genes (*SAPs*, *ALS3*, and *Hsp21*) are upregulated both with respect to cells from biofilms on polystyrene and polycarbonate. Conversely, from Figure 9B, it can be seen that *CYR1* gene is upregulated in cells from biofilm on polystyrene with respect to cells from biofilm on polycarbonate while other genes expression may be either not significantly different (*Hsp1* and *SAP3*) or upregulated in biofilms on polystyrene (*ALS3*) or upregulated in biofilms on polycarbonate (*SAP1* and *SAP2*).

**Figure 10:**
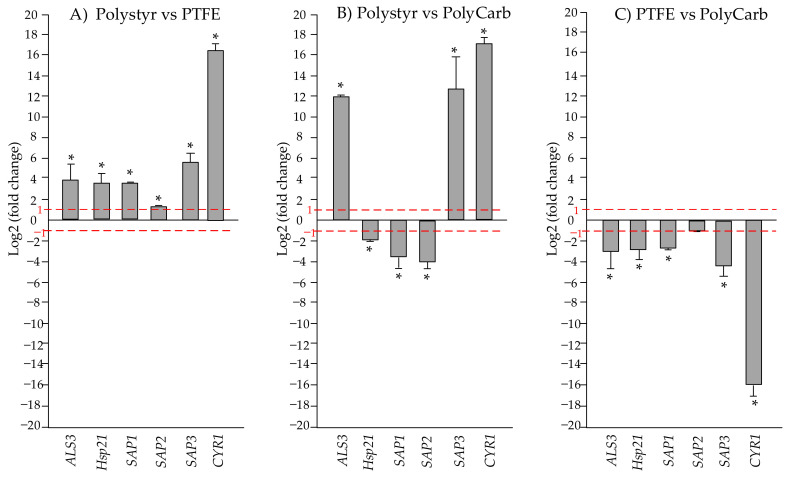
Mean relative mRNA expression levels of virulence and stress response genes of *C. tropicalis* DSM 11951. (**A**) polystyrene vs. polytetrafluoroethylene (PTFE); (**B**) polystyrene vs. polycarbonate; (**C**) PTFE vs. polycarbonate. Fold change was calculated according to the formula $\mathrm{fold}\;\mathrm{change}\;=2^{-\Delta \Delta \mathrm{Ct}}$ by using Ct (threshold cycle number) generated by the qRT-PCR system. Reported fold changes are the means of three replicate experiments. Fold changes significantly different from 1 (*p* value < 0.05) are marked by an asterisk. Red broken lines indicate fold change thresholds of 2.0 and 0.5, respectively.

Figure 10 is strictly analogous to Figure 9, except that it presents results of real-time qRT-PCR relative gene expression quantitation for *C. tropicalis* DSM 11951 strain. As seen in Figure 10A,C, *CYR1* gene follows the same trend of other tested genes, being downregulated in cells from biofilms grown on PTFE both with respect to biofilms on polystyrene and polycarbonate.

## 3. Discussion

*Candida* is one of the most common cause of infections in hospitalized patients. This frequency is the result of the ability of *Candida* species to form biofilm on biomedical devices [12]. However, despite their importance, the investigation and study of biofilms in materials sciences have not been conducted enough or many aspects related to the biofilm formation on different substrates were not considered [13]. Among the underestimated aspects, the effect of substrate on the metabolic profile of biofilm forming microorganisms may be relevant because products of cell metabolism reflect its physiological state [14,15]. Microorganisms are able to adjust their metabolism depending on many factors, such as temperature, nutrient availability, host, and the type of substrate [16,17].

To the best of our knowledge, this is the first report in which intracellular metabolites and the expression of a set of selected genes of *C. tropicalis* grown on different substrates of biomedical relevance (i.e., polystyrene, polycarbonate, and PTFE) are compared.

First, the adherent total biomass in biofilms grown on each of the three surfaces materials was quantified. Our previous article had shown that microbial biofilms grew differently depending on different surfaces textures, implying different interactions between microorganisms and initial attachment and expansion of biofilm, both in static and dynamic growth manners [18]. Polystyrene, polycarbonate, and PTFE have been studied here and data suggest that both *C. tropicalis* clinical isolate and DSM 11951 strain were resistant, surface adhering, and hydrophobic. Biofilms are most efficiently formed on polycarbonate and polystyrene surfaces while biofilm-forming ability of *C. tropicalis* on PTFE surfaces is significantly reduced.

Subsequently, the effect of each substrate on the biofilm formation of *C. tropicalis* was investigated using a GC-MS-based metabolomic fingerprint strategy, which focuses on the analysis of intracellular metabolites.

A total of 36 metabolites were identified in the intracellular extracts of the clinical isolate of *C. tropicalis* grown on polystyrene, polycarbonate, and PTFE (see Table 1).

It was observed that the metabolic pathways affected by the substrate are essentially the central carbon metabolism (i.e., glycolysis/gluconeogenesis and citrate cycle) and amino acids and lipids metabolism.

The exploration of the data in Figure 7 and Figure 8 immediately discloses that the deepest metabolic difference between cells from biofilms grown on polystyrene or PTFE and cells from biofilms grown on polycarbonate is due to the dramatic upregulation of a number of fatty acids (i.e., 9-hexadecenoic acid, 9-octadecenoic acid, 9,12-octadecadienoic acid, palmitic acid, and stearic acid) in cells from biofilms grown on polycarbonate. This observation forces attention toward citrate, an intermediate of the tricarboxylic acid cycle (TCA) that provides a bridge between glycolysis, TCA cycle, and fatty acids metabolism.

TCA cycle plays a crucial role in metabolism. Many nutrients, such as amino acids and sugars, can be metabolized into TCA intermediates and enter this cycle, but intermediates can be removed from the cycle for the use in biosynthetic pathways.

Citrate can be exported from the TCA cycle to the cytosol and once in the cytosol it is broken down into acetyl-CoA and oxaloacetate. In the cytosol, acetyl-CoA is further processed to malonyl-CoA, which can be incorporated into fatty acids. Conversely, within the TCA cycle, citrate is produced by the condensation of acetyl-CoA, which may be derived from glycolysis, with oxaloacetate deriving from the previous turn of the TCA cycle [19].

Removal (in *C. tropicalis* cells from biofilm grown on polycarbonate) of the citrate from the TCA cycle to produce fatty acids may be the key event that allows explaining most of the observed metabolic changes between cells from biofilms grown on different substrates.

The first evidence is that citrate itself is a conserved (detected) metabolite only in extracts of cells from polystyrene and PTFE biofilms and, as a consequence, it is downregulated in cells from polycarbonate biofilms. This is what is expected if citrate is removed and employed for fatty acids production.

Second, the levels of metabolites that can be sucked into the TCA cycle are also expected to be reduced in cells from biofilm grown on polycarbonate as a side effect of the leakage of citrate from the TCA cycle and, ultimately, they may contribute to the production of fatty acids. This may explain why lactic acid, which can participate in the TCA cycle indirectly by being converted to pyruvate, the precursor of oxaloacetate, is not a conserved metabolite in extracts from cells grown on polycarbonate. This explanation can be extended to most identified amino acids, including alanine, serine, aspartic acid, threonine, glutamic acid, and pyroglutamic acid, which are present at reduced levels in cells from biofilms grown on polycarbonate, both with respect to biofilms grown on polystyrene and on PTFE (see Figure 7 and Figure 8).

Third, withdrawal of citrate from the TCA cycle is expected to have consequences on the level of intermediates that come after citrate in the TCA cycle and that, analogously to citrate, are expected to be depauperated in cells from biofilm grown on polycarbonate. In accordance with this prevision, we observed downregulation in cells from biofilm grown on polycarbonate, of malate and fumarate, although succinate, which is not detected in the PolyStyr class, appears slightly upregulated (in the PolyCarb class with respect to PolyStyr class).

Finally, we suggest that the assortment of saturated and unsaturated fatty acids produced by *C. tropicalis* during biofilm development on the polycarbonate substrate may assist to finely modulate the fluidity and hydrophobicity of the cell membrane in order to improve adhesion and fasten biofilm growth. This may be an important factor providing an explanation for the relatively efficient adhesion and growth of *C. tropicalis* biofilms on polycarbonate surfaces with respect to other surface materials.

Comparing the intracellular metabolites of *C. tropicalis* on different substrates, it was found that trehalose (ID_62) is a conserved (detected) metabolite only in biofilms grown on PTFE (Table 1 and Figure 5A). This sugar has relevant dynamic metabolic functions in fungi representing, first, an important reserve of carbohydrates because it is produced when glucose is abundant in the environment. However, trehalose is a nonreducing disaccharide constituted by two glucose subunits that can be degraded to glucose under glucose deficient conditions. Therefore, the accumulation of trehalose in cells may be related to the reduced metabolic activity and growth rate of *C. tropicalis* biofilms grown on PTFE.

Vitamin B6 appears to be upregulated when the levels of intracellular metabolites from polystyrene and PTFE biofilms were compared to the ones from polycarbonate. A possible explanation of this can be obtained by considering that both trehalose and vitamin B6 are reported for their capability to act as cellular protectors against a variety of stressors. These metabolites may be considered as biomarkers of the stress exposure of the fungus. Hence, the different accumulation of these metabolites in cells from biofilms grown on different surfaces may represent a signal of the unfavorable environment for the biofilm formation caused by the composition or texture of the substrate material.

In the last decades, considerable interest has been devoted to microorganisms capable of efficiently producing d-arabitol from glucose. Yeasts of the genus *Pichia*, *Candida*, *Saccharomices*, *Zigoaccharomyces,* and *Debaryomices* are examples of microorganisms capable of producing significant amounts of d-arabitol, when grown on glucose, as the only carbon source [20]. In the case of *C. albicans*, d-arabitol is synthesized by dephosphorylating and by reducing the pentose phosphate pathway intermediate d-ribulose-5-phosphate [21]. Since d-arabitol is strictly connected to xylitol (d-arabitol is the 2-epimer of xylitol), it is possible to genetically modify d-arabitol-producing yeasts and to create engineered microorganisms capable of producing, in a single fermentation process, xylitol from glucose through a glucose-d-arabitol-d-xylulose-xylitol pathway [20]. After all, the economic advantage of converting microbial d-arabitol into xylitol depends on the fact that, although both d-arabitol and xylitol have similar properties and are both employed as sucrose substitute in a wide variety of foods, xylitol is the most valuable product because of a number of additional favorable physiological and medical functions. Furthermore, production of xylitol via the glucose-d-arabitol-d-xylulose-xylitol pathway is a convenient alternative to the microbiological process of reduction of d-xylose to xylitol. While glucose is a cheap substrate readily available in a high degree of purity, obtaining d-xylose in a form suitable for yeast fermentation is a problem since inexpensive xylose sources contain impurities that inhibit yeast growth [22].

Our data confirm that *C. tropicalis* in biofilms is an efficient producer of d-arabitol from glucose.

In fact, all GC-MS chromatograms collected throughout this study are dominated by an impressively intense chromatographic peak which, on average, accounts for about 40% of the total ionic current of the chromatogram.

The average retention time of this peak in different samples translates into a Kovats non-isothermal retention index (RI) of 1761 ± 3, which matches well with the literature RI of authentic d-arabitol 5TMS derivative (RI = 1761) determined on a DB5-MS column (which is virtually identical to the HP-5MS column employed by us) [23]. Mass spectra extracted at the apex of corresponding chromatographic peaks from different samples match the MS spectrum of d-arabitol 5 TMS derivative in the NIST14 mass spectral library, with an average match factor of 870 (out of 1000, which indicates perfect correspondence between the analyte and library mass spectrum) and an average reverse match of 890. Overall, it is completely justified to associate this well-developed chromatographic peak, which appears in all samples, with d-arabitol which, then, is by far the most abundant identified intracellular metabolite produced by *C. tropicalis* under all investigated conditions. Nevertheless, since d-arabitol is present at relatively high and comparable levels in all analyzed samples, it does not contribute to the discrimination between classes (Tables S1–S3).

On the contrary, a second sugar alcohol, d-mannitol (ID_26), which is present only in extracts from cells grown on polystyrene and PTFE and which is not detected in extracts from cells grown on polycarbonate, plays a primary role for discrimination between classes (see Figure 5). From one side, d-mannitol is upregulated in PTFE biofilms with respect to polystyrene biofilms (see Figure 6) and, on the other, it is downregulated in polycarbonate biofilms both with respect to PTFE and polystyrene biofilms (see Figure 7 and Figure 8). Since several cellular physiological functions (e.g., storage and transport of carbohydrates, protection from environmental and osmotic stresses, control of the NADP/NADPH ratio, and storage of metabolic reducing equivalents) are associated with d-mannitol in fungal cells, we suggest that the relatively high level of d-mannitol in cells from biofilms grown on PTFE is a further indicator (together with the above mentioned upregulation of trehalose and vitamin B6) of relatively unfavorable conditions for biofilm formation and growth on the PTFE surface, which, obviously, is chemically different from polystyrene and polycarbonate surfaces (Figure 1).

To gather further information on the impact of the substrate on biofilm formation by *C. tropicalis*, the expression of six selected genes (i.e., *ALS3*, *Hsp21*, *SAP1*, *SAP2*, *SAP3*, and *CYR1*) was evaluated for both strains.

*ALS* family of genes (which encode for adhesins that help microorganism to bind to host cells), *SAP* genes (which encode secreted aspartyl proteinases that are extracellular hydrolytic enzymes participating to the mechanism of aggression against host tissues), and *CYR* genes (which are involved in hyphal production) are essential virulence attributes of *Candida* species. On the other side, *Hsp21* gene encodes for a small heat shock protein that is crucial to resist specific stressors, including thermal and oxidative stress. Then, the activation of these genes determines the production of several molecules that may impact biofilm development on different substrates.

According to data in Figure 9, the expression of the hyphae-related gene *CYR1* appears to be downregulated in cells from biofilm grown on PTFE compared with cells grown on polycarbonate or polystyrene. The downregulation of this gene in biofilms on PTFE is in accordance with the evidence that PTFE is the substrate with the lowest biofilm adhesion among the evaluated substrates (Figure 1). In fact, the activation of the gene *CYR1* seems to be particularly relevant during the yeast-to-hyphal growth transition. This event requires the rapid activation of the adenylyl cyclase to generate cAMP production, subsequent activation of cAMP-responsive protein kinase A subunits, and increased transcription of genes involved in or associated with hyphal growth [24,25]. The hypha-associated adhesin Als3 is also involved in the adhesion of the fungus, but our data reveal that *ALS3* gene is inversely regulated with respect to *CYR1* gene in both pairwise comparisons of polystyrene and PTFE and PTFE and polycarbonate.

Upregulation of *Hsp21* gene, on one side, and upregulation of trehalose and vitamin B6, on the other side, in cells from biofilm grown on PTFE with respect to cells grown on polystyrene or polycarbonate, may both contribute to the answer of the microorganism to the putatively unfavorable conditions for biofilm formation on PTFE.

*C. tropicalis* is thought to possess at least four *SAP* genes [26] that encode secreted aspartyl proteinases (Saps), which are virulence factors that can act on a diversity of substrates, allowing for a variety of host proteins to be used as a nitrogen source. Nevertheless, in a recent investigation, it was reported that, in *Candida albicans*, *SAP* genes transcription increases under stress conditions induced in vitro by the presence of fluconazole (an antifungal drug), and it was suggested that upregulation of *SAP* genes may also represent a stress response factor [27]. This interpretation of *SAP* genes activation may provide understanding of the observed upregulation of these virulence genes during *C. tropicalis* clinical isolate biofilm development on the PTFE surface compared to polystyrene and polycarbonate surfaces, considering that *SAPs* upregulation takes place under the same conditions in which upregulation of *Hsp21* gene, trehalose, vitamin B6, and D-mannitol is observed.

## 4. Materials and Methods

### 4.1. C. tropicalis Strains and Growth Condition

In this study, *C. tropicalis* DSM 11951 [28] and a *C. tropicalis* clinical isolate from systemic infection [29], molecularly identified ≥98% and compared with a reference strain *C. tropicalis* EF216862.1, were used.

Stock cultures were maintained in glycerol at −80 °C prior to use. Microbial inocula were prepared from overnight cultures at 37 °C of single colonies into Tryptone Soya Broth (TSB) (OXOID, Basingstoke, UK) with 0.1% of glucose. Cultures were washed twice using phosphate buffered saline (PBS) (OXOID) and standardized to 10^6^ cells mL^−1^ for next experiments.

### 4.2. Development and Quantification of Biofilms

To evaluate the ability of *C. tropicalis* to form biofilms on different surfaces, coupons in polystyrene, polycarbonate, and Teflon (BioSurface Technologies Corp., Bozeman, MO, USA) were used. Briefly, 1 mL of microbial culture (10^6^ cells mL^−1^) was added to the wells of 24-well microplate containing sterile coupons and incubated at 37 °C for 72 h under static conditions.

For enumerating colony-forming units (CFU) of biofilm, the coupons surface was washed to eliminate the not adhered cells with 1 mL of PBS and scraped using a sterile scraper. Then, biofilm cells were suspended in 10 mL of PBS, serially diluted, and plated on Rose Bengal agar (Microbiol Diagnostic). The plates were incubated at 37 °C for 24 h before counting. Biofilm population density was recorded as log10 colony forming units per unit surface area (i.e., log10(fungal density) = log10(CFU·cm^−2^)).

Statistical analyses were performed using GraphPad Prism Software (version 8.02 for Windows, GraphPad Software, La Jolla, CA, USA). All experiments were performed in triplicate. The results were reported as a log10(mean) ± standard deviation. Data were assessed considering the analysis of variance (ANOVA) and multiple comparison was applied to different selected materials (Tukey’s, *p* value < 0.05).

### 4.3. GC-MS Analysis of Intracellular Metabolites

#### 4.3.1. Sample Preparation

Intracellular metabolites of both strains of *C. tropicalis* were extracted as previously described with slight modifications [30,31]. Briefly, following the cultivation, biofilm cells were rapidly washed with ice-cold ultrapure sterile water to remove the medium, and then the cells were cooled at −80 °C for quick quenching. Each sample was homogenized with 75 µL of cold methanol:water (60:40) and incubated for 30 min at −20 °C. After centrifugation for 20 min at 18,000× *g* and 4 °C, the upper organic layer was transferred to a new tube. The second homogenization with 200 µL of methanol:chloroform (3:1) and centrifugation were performed subsequently. All the upper organic phase of the second extraction were combined with the first supernatant and then dried with a stream of nitrogen. The residues were derivatized with *N*,*O*-bis(trimethylsilyl)-trifluoroacetamide (BSTFA) (Fluka, Buchs, Switzerland) as previously described [32].

#### 4.3.2. GC-MS Analysis

Trimethylsilyl derivatives were analyzed by an Agilent 6850 GC (Milan, Italy), equipped with an HP-5MS capillary column (5% phenyl methyl poly siloxane stationary phase), coupled to an Agilent 5973 Inert MS detector operated in the full scan mode (*m*/*z* 29–550) at a frequency of 3.9 Hz and with the EI ion source and quadrupole mass filter temperatures kept, respectively, at 200 and 250 °C. Helium was used as carrier gas at a flow rate of 1 mL·min^−1^. The injector temperature was 250 °C and the temperature ramp raised the column temperature from 70 to 280 °C: 70 °C for 1 min; 10 °C·min^−1^ until reaching 170 °C; and 30 °C·min^−1^ until reaching 280 °C. Then, it was held at 280 °C for 5 min. The solvent delay was 4 min.

#### 4.3.3. Data Processing and Statistical Analysis

Cell extracts, after derivatization with BSTFA, were submitted to GC-MS analysis and, overall, we collected two datasets (one dataset for each of the two *C. tropicalis* strains) each comprising 18 GC-MS data files (GC-MS chromatograms).

The clinical and DSM 11951 strains datasets were processed separately as described below.

GC-MS data in a dataset are composed of three classes (or conditions) that are distinguished by the nature of the adhesion material (i.e., polystyrene, polycarbonate, and PTFE). Each of the three classes is composed of six observations (GC-MS datafiles) corresponding to three technical replicates for each of two biological replicates under the same condition.

Each GC-MS data file was processed with the National Institute of Standards and Technology (NIST) program automated mass spectral deconvolution and identification system (AMDIS) [33] which detects components (metabolites) and associates to each a retention time (RT), its Kovats index (RI), the relative abundance (RA), and the deconvoluted 70 eV EI mass spectrum (MS). For each submitted GC-MS data file, AMDIS results are incorporated in a .ELU file, which is used for further processing. Finally, AMDIS results files were presented to SpectConnect software, which performs components matching within and between classes [9]. In the context of SpectConnect, a component whose signal systematically persists in replicated observations in the same class (or condition), is designated as a “conserved component or metabolite”. In order to be retained, a component needs to be conserved in at least one of the three classes: PolyStyr, Polycarb, or PTFE. Components that do not satisfy this condition are considered as accidents or artifacts of noise and are ignored.

After processing, SpectConnect provides a cumulative library that lists conserved components identified by an ID string and other relevant information extracted from the AMDIS results files. Furthermore, and most importantly, SpectConnect provides an RA (relative abundance) matrix that has a number of rows equal to the number of observations in the submitted classes (18 in the present case) and a number of columns equal to the number of conserved metabolites. A cell in the RA matrix contains the relative abundance of the metabolite specified by the ID string in the column label in the observation specified by the row label.

The above pre-processing of the two datasets of GC-MS data collected in this study provides a cumulative library of 60 conserved metabolites and an 18 × 60 RA matrix for the DSM 11951 strain dataset. Analogously, we obtain a cumulative library of 69 conserved metabolites and an 18 × 69 RA matrix for the clinical isolate dataset.

Only a fraction of the conserved metabolites in the two datasets can be identified. In particular, we have been able to identify 35 (out of 60) metabolites in the DSM 11951 strain dataset and 36 (out of 69) metabolites in the clinical isolate dataset.

The final data matrix was submitted to multivariate statistical analyses.

To this end, the RA matrix is imported in our in-house .m script in MATLAB R2020b (Mathworks, Natick, MA, USA) [10], which has the logic for performing principal component analysis (PCA) and partial least-squares discriminant analysis (PLS-DA) multiclass comparisons. Before multivariate analysis, the RA matrix is mean centered and variance scaled. After PLS-DA analysis, variables significantly contributing to the clustering and discrimination of samples were identified according to variable influence on projection (VIP) values generated by PLS-DA processing.

Subsequently, univariate analysis, which allows comparison of metabolites one by one, was performed. Univariate analysis associates to each metabolite a fold change (FC) and *p* value that is calculated by performing a paired Student’s *t*-test. This was accomplished by importing the RA matrix in a second MATLAB .m script [10]. The script detects classes which comprise the RA matrix and asks the user to select from a list the two classes to be compared. After class selection, the script creates from the RA matrix a reduced matrix, RA* matrix, containing only observations in the two selected classes. In the present case, the RA* matrix is composed of 12 rows containing 6 replicate observations in each of the two classes to be compared.

The metabolites identification was performed by comparing their deconvoluted EI mass spectra at 70 eV with those present in the NIST 14 mass spectral library [34] and the Golm metabolome database [35,36]. Furthermore, the identification was supported by the Kovats retention index (RI) calculated for each metabolite by the Kovats equation using the standard n-alkane mixture in the rang C7–C40 (Sigma-Aldrich, Saint Louis, MO, USA) [14,37].

### 4.4. RNA Extraction and Expression Profiling by Real-Time qRT-PCR

After 72 h development on coupons in polystyrene, polycarbonate, and Teflon, biofilms of DSM 11951 and clinical strains of *C. tropicalis* were collected in a 2 mL tube, kept on ice immediately, and were further homogenized in TRIzol (Invitrogen, Paisley, UK) using a TissueLyser II (Qiagen, Valencia, CA, USA) and steal beads of 7 mm diameter (Qiagen, Valencia, CA, USA). Total RNA was extracted and purified using Direct-zol™ RNA Miniprep Plus Kit (ZYMO RESEARCH, Irvine, CA, USA). The amount of total RNA extracted was estimated by the absorbance at 260 nm and the purity by 260/280 and 260/230 nm ratios, using a NanoDrop spectrophotometer 2000 (Thermo Scientific Inc., Waltham, MA, USA) to exclude the presence of proteins, phenol, and other contaminants [38]. For each sample, 1000 ng of total RNA was retrotranscribed with an iScript™ cDNA Synthesis kit (Bio-Rad, Milan, Italy) following the manufacturer’s instructions. Afterward, the variations in gene expression of three virulence genes (*SAP1*, *SAP2*, and *SAP3*), one adhesion gene (*ALS3*), one gene involved in hyphal formation (*CYR1*), one gene involved in stress response (*Hsp21*), and normalizer *ACT1* (see Table S4) were evaluated. Undiluted cDNA was used as a template in a reaction containing a final concentration of 0.3 mM for each primer and 1 × SensiFAST^TM^ SYBR Green master mix (total volume of 10 µL) (Meridiana Bioline, Cincinnati, OH, USA). PCR amplifications were performed in a AriaMx Real-Time PCR instrument (Agilent Technologies, Inc., Santa Clara, CA, USA) using the following thermal profile: 95 °C for 10 min, one cycle for cDNA denaturation; 95 °C for 15 s and 60 °C for 1 min, 40 cycles for amplification; 95 °C for 15 s; one cycle for final elongation; and one cycle for melting curve analysis (from 60 to 95 °C) to verify the presence of a single product. Each assay included a no-template control for each primer pair. To capture intra-assay variability, all real-time qPCR reactions were conducted in triplicate. The PCR reactions efficiency of all assays, determined from the data generated from serial dilutions over several orders of magnitude of the target and normalizer templates, was comprised between 96% and 103% so that fold changes could accurately be evaluated by employing the 2^−^^ΔΔCt^ method. Fluorescence was measured using Agilent Aria 1.7 software (Agilent Technologies, Inc.).

## 5. Conclusions

In this study, the production of intracellular metabolites and the expression of a set of selected genes of *C. tropicalis* grown on three different substrates of biomedical relevance were investigated, with the aim to improve the overall understanding on the fungal adhesion on different materials. Medical devices are frequently contaminated by *C. tropicalis,* and the pathogenicity of this fungus is linked to its great ability to adhere to diverse material forming biofilm.

Our data show that *C. tropicalis* is able to form biofilm on polystyrene, polycarbonate, and PTFE after 72 h of incubation, even if biofilms are most efficiently formed on polycarbonate and polystyrene. The different accumulation of intracellular metabolites and different expression of virulence genes observed in cells from biofilms grown on different surfaces may represents an answer of the microorganism to the environment caused by the composition and texture of substrate materials.

## Figures and Tables

**Table 1:** Results of multiclass PLS-DA analysis in Figure 4.

Name	ID_##	VIP	In Class
Trehalose, 8 TMS (RI = 2815)	ID_62	1.671	[3]
Glycerol monostearate, 2 TMS (RI = 2794)	ID_31	1.532	[1,2]
l-Monopalmitin, 2 TMS (RI = 2599)	ID_30	1.515	[1,2,3]
Succinic acid, 2 TMS (RI = 1321)	ID_37	1.338	[2,3]
d-Mannitol, 6 TMS (RI = 1968)	ID_26	1.326	[1,3]
l-Tyrosine, 3 TMS (RI = 1962)	ID_25	1.156	[1,2,3]
l-Lysine, 3 TMS (RI = 1722)	ID_34	1.148	[1,3]
Phosphoric acid, 3 TMS (RI = 1292)	ID_8	1.118	[1,2,3]
Methyl bis(trimethylsilyl) phosphate (RI = 1199)	ID_67	1.058	[3]
l-Proline, 2 TMS (RI = 1310)	ID_9	0.997	[1,2,3]
Tromethamine *N*-acetyl, 3 TMS (RI = 1614)	ID_19	0.996	[1,3]
Tromethamine, 4 TMS (RI = 1537)	ID_15	0.986	[1,2,3]
Lactic Acid, 2 TMS (RI = 1079)	ID_3	0.962	[1,3]
Leucine, 2 TMS (RI = 1284)	ID_32	0.945	[1,2,3]
Arachidic acid, TMS (RI = 2442)	ID_29	0.926	[1]
Vitamin B6, 3 TMS (RI = 1923)	ID_24	0.921	[1,3]
l-Aspartic acid, 3 TMS (RI = 1544)	ID_16	0.918	[1,3]
Fumaric acid, 2 TMS (RI = 1353)	ID_10	0.912	[1,3]
9-Hexadecenoic acid, (Z)-, TMS (RI = 2035)	ID_46	0.908	[2]
l-Threonine, 3 TMS (RI = 1402)	ID_12	0.893	[1,2,3]
Phosphoric acid, bis(trimethylsilyl) 2,3-bis[(trimethylsilyl)oxy]propyl ester (RI = 1793)	ID_22	0.891	[1,2,3]
l-Alanine, 2 TMS (RI = 1120)	ID_4	0.889	[1,2,3]
l-5-Oxoproline, 2 TMS (RI = 1546)	ID_17	0.885	[1,2,3]
l-Glutamic acid, 3 TMS (RI = 1640)	ID_20	0.884	[1,2,3]
Malic acid, 3 TMS (RI = 1504)	ID_14	0.883	[1,2,3]
Citric acid, 4 TMS (RI = 1846)	ID_36	0.859	[1,3]
l-Serine, 3 TMS (RI = 1374)	ID_11	0.833	[1,2,3]
Stearic acid, TMS (RI = 2246)	ID_28	0.824	[1,2,3]
l-Phenylalanine, 2 TMS (RI = 1646)	ID_39	0.793	[2]
Glycine, 2 TMS (RI = 1133)	ID_5	0.788	[1,2,3]
9,12-Octadecadienoic acid (Z,Z)-, TMS (RI = 2237)	ID_48	0.751	[2]
l-Valine, 2 TMS (RI = 1229)	ID_7	0.747	[1,2,3]
Palmitic Acid, TMS (RI = 2050)	ID_27	0.747	[1,2,3]
9-Octadecenoic acid, (E)-, TMS (RI = 2237)	ID_49	0.723	[2]
d-Arabitol, 5 TMS (RI = 1761)	ID_21	0.609	[1,2,3]

Identified metabolites are sorted according to descending VIP scores. The array in the column labeled “In Class” indicates ordinately the class membership of metabolites (PolyStyr = 1; PolyCarb = 2; and PTFE = 3). RI represents Kovats retention index and TMS is the trimethylsilyl function, (CH_3_)_3_Si-.

## Data Availability

Not applicable.

## Supplementary Materials

The following are available online at https://www.mdpi.com/article/10.3390/ijms22169038/s1.
