# Supplementary material for: An Integrated Analysis of Intracellular Metabolites and Virulence Gene Expression during Biofilm Development of a Clinical Isolate of Candida tropicalis on Distinct Surfaces
Source: Int J Mol Sci. 2021 Aug 21;22(16):9038. doi: 10.3390/ijms22169038 (PMC8396647; doi:10.3390/ijms22169038)
Supplement: Supplementary file 1 [file ijms-22-09038-s001.zip › ijms-1339764-supplementary.pdf]

Supplementary Materials

An Integrated Analysis of Intracellular Metabolites and Virulence Gene Expression during Biofilm Development of a Clinical Isolate of *Candida tropicalis* on Distinct Surfaces

Maria Michela Salvatore [1], Angela Maione [2], Luisa Albarano [2], Elisabetta de Alteriis [2], Federica Carraturo [2], Anna Andolfi [1,3], Francesco Salvatore [1], Emilia Galdiero [2,*], Marco Guida [2]

[1] Department of Chemical Sciences, University of Naples 'Federico II', via Cinthia, 80126 Naples, Italy; mariamichela.salvatore@unina.it (M.M.S.); andolfi@unina.it (A.A.); frsalvat@unina.it (F.S.)
[2] Department of Biology, University of Naples 'Federico II', via Cinthia, 80126 Naples, Italy; egaldier@unina.it (E.G.); angela.maione@unina.it (A.M.); luisa.albarano@unina.it (L.A.); dealteriis@unina.it (E.d.A.); federica.carraturo@unina.it (F.C.); marco.guida@unina.it (M.G.)
[3] BAT Center—Interuniversity Center for Studies on Bioinspired Agro-Environmental Technology, University of Naples 'Federico II', 80055 Naples, Italy
* Correspondence: egaldier@unina.it Tel.: +39-081-679182

Table S1. Results of univariate comparison between PolyStyr and PTFE biofilm classes. VIP scores reported in column 3 have been obtained after PLS-DA analysis shown in Figure 5B. Up = Upregulated in PolyStyr class; Down = Downregulated in PolyStyr class. Identified metabolites can be tracked back to identified metabolites in Table 1 through the associated ID_## string.

Name	ID_##	VIP	p_Value	Fold Change	Trend
Trehalose, 8TMS	ID_62	1.512	0.0001884	17.88	Down
D-Mannitol, 6TMS	ID_26	1.500	0.0002933	2.14	Down
Glycerol monostearate, 2TMS	ID_31	1.471	0.0003684	5.793	Up
Tromethamine, 4TMS	ID_15	1.444	0.001048	1.491	Up
1-Monopalmitin, 2TMS	ID_30	1.414	0.001973	3.451	Up
Succinic acid, 2TMS	ID_37	1.396	0.003483	4.23	Down
Phosphoric acid, 3TMS	ID_8	1.360	0.00489	1.941	Up
L-Aspartic acid, 3TMS	ID_16	1.256	0.01747	1.374	Up
Palmitic Acid, TMS	ID_27	1.167	0.03538	1.477	Up
L-Lysine, 3TMS	ID_34	1.162	0.0413	4.39	Up
L-Proline, 2TMS	ID_9	1.122	0.04607	1.57	Up
Stearic acid, TMS	ID_28	1.013	0.08639	1.376	Up
Tromethamine N-acetyl, 3TMS	ID_19	1.036	0.09284	1.254	Down
L-Tyrosine, 3TMS	ID_25	0.971	0.1014	1.503	Down
Arachidic acid, TMS	ID_29	0.887	0.1186	2.608	Up
Malic acid, 3TMS	ID_14	0.935	0.1231	1.338	Up
Phosphoric acid, bis(trimethylsilyl) 2,3-bis[(trimethylsilyl)oxy]propyl ester	ID_22	0.921	0.1304	1.117	Up
Methyl bis(trimethylsilyl) phosphate	ID_67	0.896	0.1517	2.408	Down
L-5-Oxoproline, 2TMS	ID_17	0.884	0.1551	1.632	Up
L-Alanine, 2TMS	ID_4	0.900	0.163	1.13	Up
Citric acid, 4TMS	ID_36	0.830	0.192	1.711	Up
Leucine, 2TMS	ID_32	0.779	0.2312	1.279	Up
Lactic Acid, 2TMS	ID_3	0.788	0.2567	1.262	Down
L-Glutamic acid, 3TMS	ID_20	0.695	0.3027	1.098	Up
Vitamine B6, 3TMS	ID_24	0.664	0.3122	1.478	Down
Glycine, 2TMS	ID_5	0.599	0.431	1.421	Up
Serine, 3TMS	ID_11	0.556	0.436	1.099	Up
D-Arabitol, 5TMS	ID_21	0.618	0.4942	1.263	Down
L-Valine, 2TMS	ID_7	0.508	0.5171	1.108	Up
Fumaric acid, 2TMS	ID_10	0.412	0.5942	1.076	Down
L-Threonine, 3TMS	ID_12	0.442	0.6882	1.061	Down
L-Phenylalanine, 2TMS	ID_39	0.159	0.8769	1.128	Down

Table S2. Results of univariate comparison between PolyStyr and PolyCarb biofilm classes. VIP scores reported in column 3 have been obtained after PLS-DA analysis shown in Figure 5C. Up = Upregulated in PolyStyr class; Down = Downregulated in PolyStyr class. Metabolites can be tracked back to identified metabolites in Table 1 through the associated ID_## string.

Name	ID_##	VIP	p-Value	Fold Change	Trend
Vitamine B6, 3TMS	ID_24	1.141	1.80E-07	24.23	Up
D-Mannitol, 6TMS	ID_26	1.139	4.71E-07	26.74	Up
L-Glutamic acid, 3TMS	ID_20	1.134	1.95E-06	7.037	Up
Tromethamine, 4TMS	ID_15	1.134	2.25E-06	7.731	Up
Phosphoric acid, bis(trimethylsilyl) 2,3-bis[(trimethylsilyl)oxy]propyl ester	ID_22	1.132	3.11E-06	5.842	Up
L-Alanine, 2TMS	ID_4	1.129	8.32E-06	19.81	Up
L-Aspartic acid, 3TMS	ID_16	1.125	1.63E-05	37.74	Up
9-Hexadecenoic acid, (Z)-, TMS	ID_46	1.127	1.66E-05	284.9	Down
Tromethamine N-acetyl, 3TMS	ID_19	1.115	4.90E-05	47.16	Up
L-Proline, 2TMS	ID_9	1.096	0.0001976	3.425	Up
Fumaric acid, 2TMS	ID_10	1.101	0.0002055	15.86	Up
Phosphoric acid, 3TMS	ID_8	1.092	0.0002459	6.733	Up
Serine, 3TMS	ID_11	1.09	0.0002681	2.374	Up
L-Threonine, 3TMS	ID_12	1.087	0.000326	6.863	Up
Glycerol monostearate, 2TMS	ID_31	1.065	0.0005854	3.687	Up
L-Phenylalanine, 2TMS	ID_39	1.051	0.001145	4.095	Down
Malic acid, 3TMS	ID_14	1.055	0.001221	4.897	Up
Succinic acid, 2TMS	ID_37	1.04	0.002126	4.297	Down
Leucine, 2TMS	ID_32	1.037	0.002684	1.939	Down
Citric acid, 4TMS	ID_36	1.034	0.002882	36.7	Up
Lactic Acid, 2TMS	ID_3	1.028	0.003494	17.47	Up
1-Monopalmitin, 2TMS	ID_30	1.018	0.003576	2.649	Up
Stearic acid, TMS	ID_28	0.991	0.005386	87.32	Down
L-5-Oxoproline, 2TMS	ID_17	0.953	0.01129	6.664	Up
L-Valine, 2TMS	ID_7	0.941	0.01352	1.836	Up
L-Lysine, 3TMS	ID_34	0.944	0.01539	30.27	Up
9,12-Octadecadienoic acid (Z,Z)-, TMS	ID_48	0.895	0.02407	4289	Down
Palmitic Acid, TMS	ID_27	0.887	0.02608	63.07	Down
Arachidic acid, TMS	ID_29	0.901	0.02667	5.934	Up
9-Octadecenoic acid, (E)-, TMS	ID_49	0.842	0.03767	2345	Down
Glycine, 2TMS	ID_5	0.689	0.1044	1.559	Down
L-Tyrosine, 3TMS	ID_25	0.284	0.6991	1.113	Up
D-Arabitol, 5TMS	ID_21	0.208	0.8781	1.057	Down

Table S3. Results of univariate comparison between PTFE and PolyCarb biofilm classes. VIP scores reported in column 3 have been obtained after PLS-DA analysis shown in Figure 5D. Up = Upregulated in PTFE class; Down = Downregulated in PTFE class. Metabolites can be tracked back to identified metabolites in Table 1 through the associated ID_## string.

Name	ID_##	VIP	p-Value	Fold Change	Trend
L-Alanine, 2TMS	ID_4	1.149	4.50E-08	17.53	Up
Lactic Acid, 2TMS	ID_3	1.145	3.77E-07	29.54	Up
Phosphoric acid, bis(trimethylsilyl) 2,3-bis[(trimethylsilyl)oxy]propyl ester	ID_22	1.145	6.86E-07	5.231	Up
Malic acid, 3TMS	ID_14	1.142	1.26E-06	3.659	Up
Tromethamine, 4TMS	ID_15	1.140	1.80E-06	5.184	Up
Phosphoric acid, 3TMS	ID_8	1.139	2.23E-06	3.469	Up
Fumaric acid, 2TMS	ID_10	1.141	4.63E-06	22.86	Up
L-Aspartic acid, 3TMS	ID_16	1.135	5.68E-06	27.74	Up
Tromethamine N-acetyl, 3TMS	ID_19	1.135	6.36E-06	79.25	Up
D-Mannitol, 6TMS	ID_26	1.135	6.93E-06	76.69	Up
L-5-Oxoproline, , 2TMS	ID_17	1.129	1.50E-05	4.083	Up
9-Hexadecenoic acid, (Z)-, TMS	ID_46	1.131	1.66E-05	381.8	Down
L-Glutamic acid, 3TMS	ID_20	1.126	2.31E-05	6.411	Up
L-Threonine, 3TMS	ID_12	1.120	4.93E-05	7.284	Up
Leucine, 2TMS	ID_32	1.111	0.0001643	2.48	Down
Trehalose, 8TMS	ID_62	1.107	0.0001884	17.88	Up
L-Tyrosine, 3TMS	ID_25	1.083	0.0005107	1.673	Up
Serine, 3TMS	ID_11	1.075	0.0007716	2.161	Up
L-Valine, 2TMS	ID_7	1.053	0.001654	1.657	Up
L-Phenylalanine, 2TMS	ID_39	1.025	0.002859	3.693	Down
Stearic acid, TMS	ID_28	1.000	0.005307	120.1	Down
Glycine, 2TMS	ID_5	0.959	0.007792	2.251	Down
Vitamine B6, 3TMS	ID_24	0.955	0.0154	48	Up
9,12-Octadecadienoic acid (Z,Z)-, TMS	ID_48	0.891	0.02406	5748	Down
Palmitic Acid, TMS	ID_27	0.885	0.02557	93.12	Down
Methyl bis(trimethylsilyl) phosphate	ID_67	0.891	0.02601	5.282	Up
L-Proline, 2TMS	ID_9	0.899	0.02836	2.181	Up
Citric acid, 4TMS	ID_36	0.878	0.02969	28.75	Up
L-Lysine, 3TMS	ID_34	0.855	0.03681	9.24	Up
9-Octadecenoic acid, (E)-, TMS	ID_49	0.848	0.03767	3143	Down
1-Monopalmitin, 2TMS	ID_30	0.887	0.04087	1.303	Down
Arachidic acid, TMS	ID_29	0.691	0.1341	3	Up
Xylitol, 5TMS	ID_21	0.614	0.1767	1.195	Up
Succinic acid, 2TMS	ID_37	0.560	0.2766	1.122	Down
Glycerol monostearate, 2TMS	ID_31	0.460	0.4352	1.543	Down

Table S4. Name, acronym, sequence and references of used primer for *RT-qPCR.*

Name	Acronym	Primer sequence (5′-3′)	References
heat shock protein 21	hsp21_F	TTATGATTGTCGGGGTTGCT	[1]
	hsp21_R	GCCAATGGACCAGCATACCT	
secreted aspartyl proteases 1	SAP1_F	TATGACAATGTGCCAGTT	[2]
	SAP1_R	TAAAGCAGTCAAAGTCCC	
secreted aspartyl proteases 2	SAP2_F	GCTGGTTTCTGTGCTTTG	[2]
	SAP2_R	CCACGTAGGCATGTCTTA	
secreted aspartyl proteases 3	SAP3_F	ACTTGGATTTCCAGCGAAGA	[2]
	SAP3_R	AGCCCTTCCAATGCCTAAAT	
CYR 1	CYR1_F	CCAACAAACGACCAAAAGGT	[3]
	CYR1_R	TCTTGAACTGCCAGACGATG	
agglutinin-like sequences 3	ALS3_F	AGGTGCTGTAGTTGTTCTT	[2]
	ALS3_R	AGCAGTCGGGTTGAAAGG	
actin	Actin_F	GGCTGGTAGAGACTTGACCAACCATTTG	[4]
	Actin_R	GGAGTTGAAAGTGGTTTGGTCAATAC	

References

1. Wang, H.; Li, Q.; Peng, Y.; Zhang, Z.; Kuang, X.; Hu, X.; Ayepa, E.; Han, X.; Abrha, G.T.; Xiang, Q.; et al. Cellular analysis and comparative transcriptomics reveal the tolerance mechanisms of *Candida tropicalis* toward phenol. *Front. Microbiol.* **2020**, *11*, 1–19.
2. Yu, S.; Li, W.; Liu, X.; Che, J.; Wu, Y.; Lu, J. Distinct expression levels of ALS, LIP, and SAP genes in *Candida tropicalis* with diverse virulent activities. *Front. Microbiol.* **2016**, *7*, 1–10.
3. Kim, D.J.; Lee, M.W.; Choi, J.S.; Lee, S.G.; Park, J.Y.; Kim, S.W. Inhibitory activity of hinokitiol against biofilm formation in fluconazole-resistant *Candida* species. *PLoS One* **2017**, *12*, 1–11.
4. Losberger, Christophe, Ernst, J.F. Sequence of the *Candida albicans* gene encoding acti. *Nucleic Acids Res.* **1989**, *17*, 9488.
